# A Narrative Review on Efficacy and Safety of Proton Pump Inhibitors in Children

**DOI:** 10.3389/fphar.2022.839972

**Published:** 2022-02-10

**Authors:** Valeria Dipasquale, Giuseppe Cicala, Edoardo Spina, Claudio Romano

**Affiliations:** ^1^ Pediatric Gastroenterology and Cystic Fibrosis Unit, Department of Human Pathology in Adulthood and Childhood “G. Barresi”, University of Messina, Messina, Italy; ^2^ Department of Clinical and Experimental Medicine, University of Messina, Messina, Italy

**Keywords:** adverse reaction, indication, pediatrics, proton pump inhibitor, safety

## Abstract

Proton pump inhibitors (PPIs) are among the most prescribed drugs worldwide and include omeprazole, esomeprazole, lansoprazole, pantoprazole, and rabeprazole. Their use in pediatrics is approved for children older than 1 year, for the short-term treatment of symptomatic gastroesophageal reflux disease (GERD), healing of erosive esophagitis, treatment of peptic ulcer disease, and eradication of *Helicobacter pylori*. PPIs are also considered the standard of care for pediatric eosinophilic esophagitis. Despite the strict range of indications, the use of this class of molecules has increased in all pediatric age ranges. The long-term gastric acid suppression in children has been linked to increased risks of gastrointestinal and lower respiratory tract infections, bone fractures, and allergy. This study aims to provide a comprehensive overview of the mechanism of actions, use (and misuse) in infants and children, and safety of PPIs.

## 1 Introduction

Proton pump inhibitors (PPIs), such as omeprazole, esomeprazole, lansoprazole, pantoprazole, and rabeprazole, are among the most often prescribed medications in the world. Despite the limited number of indications, the use of this class of compounds has risen across the pediatric age spectrum (Levy et al., 2020). From 2000 to 2015, a 16-year register-based analysis based on Danish nationwide healthcare registries showed 212,056 filled in PPI prescriptions dispensed to 78,489 children (0–17 years old), with total annual use of PPIs among children increasing eightfold (Aznar-Lou et al., 2019). A recent Irish retrospective drug utilization study using national prescription reimbursement data found a significant and statistically significant increase in PPI prescriptions in infants under the age of 1 year over 10 years (from 1011 in 2009–2763 in 2018; *p* < .00001) (O’Reilly et al., 2020). Similar trends have been found in other European countries and in the United States (Barron et al., 2007; De Bruyne et al., 2014; Illueca et al., 2014). Furthermore, several prescriptions were discovered to be off-label due to an inadequate indication of use (i.e., outside from the license for use for children) (Levy et al., 2020; O’Reilly et al., 2020). Indeed, PPIs are often empirically prescribed for infantile reflux, functional dyspepsia, chronic cough, and asthma without documented associated gastroesophageal reflux disease (GERD). The empiric use of PPIs is being scrutinized more closely as a body of evidence accumulates about their possible dangers (Chung and Yardley, 2013; Cohen et al., 2015; Stark and Nylund, 2016; De Bruyne and Ito, 2018; Pasman et al., 2020). In this study, we will provide a comprehensive overview of the mechanism of actions, use (and misuse) in infants and older children, and safety profile of PPIs. The main goal of the present review is to re-evaluate the evidence, limitations, efficacy, and safety of PPI use in the pediatric population.

## 2 Methods

Relevant studies published over the last 20 years were identified via a PubMed/Medline search using different combinations of the following search terms: “proton pump inhibitors,” “children” and “infants.” Additional papers were identified by reviewing reference lists of relevant publications. Particular emphasis was placed on original studies investigating efficacy and/or safety issues. The summaries of product characteristics (SmPCs) were used for verification of approved indications. Non-English publications were excluded. A systematic approach to study selection was not implemented. Instead, data were extracted based on their relevance to the topic.

## 3 Pharmacological Characteristics

Effective treatment with PPIs requires an understanding of their pharmacokinetics (Ward and Kearns, 2013). The H^+^-K^+^-adenosine triphosphatase (H^+^-K^+^-ATPase) is the enzyme responsible for acid secretion by the parietal cell in the stomach, after stimulation by the binding of different ligands, such as acetylcholine, histamine, or gastrin (Litalien et al., 2005; Roche, 2006; Shin et al., 2009). Ten of the H + -K + -ATPase’s 28 cysteine (CYS) molecules are accessible to PPIs for binding. To bind to the CYS of the ATPase, PPIs must be activated, and the rate of this activation varies depending on their structures. (Litalien et al., 2005; Roche, 2006; Shin et al., 2009). PPIs are acid-sensitive weak bases that require an enteric coating to protect them from gastric acid destruction and allow absorption in the intestine. PPIs now on the market have a basic structure that connects a benzimidazole ring and a pyridine ring via a sulfinyl bond, with numerous substitutions on these rings affecting their chemistry (Roche, 2006). To chemically connect to the CYSs of the ATPase, the sulfinyl must obtain energy from the acidic environment within the parietal cell. Two protons are added to the nitrogens on either side of the sulfinyl group to activate the PPI. (Roche, 2006; Shin et al., 2009). Once active, the PPI can suppress the proton pump by forming disulfide bonds with CYS molecules on the ATPase. For the proton pump to be inhibited, the PPI must first reach the acidic site of action within the parietal cell, where it will receive the acidic activation described above (Litalien et al., 2005). The pharmacokinetics of the PPI determine the concentrations at the site of action, starting with inactive form absorption, distribution, processing by cytochrome P450 (CYP) 2C19 or CYP3A4, and clearance. Blockade of the proton pump necessitates pump activation before the PPI is eliminated from circulation, which influences the rate of absorption, latency to highest concentration, and rate of medication removal from circulation (Litalien et al., 2005). Because gastrin production after a meal is one of the most powerful H + -K + -ATPase inducers, the PPI should be taken long enough before a meal to be absorbed when the proton pump is most active. Acid secretion is stopped once the activated PPI attaches to the H + -K + -ATPase, either reversibly or permanently, long after the PPI has been removed from circulation. Because of variations in binding to the proton pump’s CYSs, omeprazole blocks acid secretion for 24 h compared to 46 h for pantoprazole (Shin et al., 2009). Because not all proton pumps are blocked after the initial dosage, it takes around 3 days to reach steady state.

## 4 Clinical Uses

### 4.1 Children and Adolescents

PPIs are licensed for use in children over the age of 1 year for the short-term treatment of symptomatic GERD, erosive esophagitis healing, peptic ulcer disease therapy, and *Helicobacter pylori* eradication. They are also considered the standard of care for pediatric eosinophilic esophagitis (EoE) (Levy et al., 2020). A systematic review including 12 studies on the use of PPIs (esomeprazole, lansoprazole, omeprazole, and pantoprazole) in children with GERD, has identified four trials in which PPIs were more effective for gastric acidity than placebo, alginic acids or ranitidine (van der Pol et al., 2011). In three of the studies, PPIs did not show significant difference from ranitidine or alginates in reducing histological alterations (van der Pol et al., 2011). A multicenter, double-blind, parallel-group study showed that rabeprazole is effective in 1- to 11-year-old children with endoscopically/histologically proven GERD randomized to receive a .5 or 1.0 mg/kg rabeprazole for 12 weeks, with further dose adjustment by weight (Haddad et al., 2013). The same authors have additionally determined the efficacy of maintenance treatment with rabeprazole in 1- to 11-year-old children suffering from GERD, showing healing maintenance in 90% of the children (Haddad et al., 2014). In children with typical GERD symptoms, current pediatric guidelines recommend a 4- to 8-week trial of PPIs (Rosen et al., 2018). Anyway, a recent study investigating the frequency of GERD in 85 toddlers with GERD symptoms found a very low incidence of GERD (3 children had abnormal reflux index at 24-hour esophageal pH monitoring, while 7 had reflux esophagitis at upper endoscopy), thus suggesting considering to be cautious with diagnostic PPI trials (Yang et al., 2019). In the pediatric age group, peptic acid disorders, such as erosive esophagitis and peptic ulcer disease, are rather uncommon conditions (Pasman et al., 2020; Ward and Kearns, 2013). The pharmacodynamics of PPIs for treatment of peptic acid disorders for children aged 1 year or older are comparable to that in adult patients (Ward and Kearns, 2013). In an international, multicenter, randomized, double-blind study conducted on children aged 1–11 years with endoscopically/histologically proven erosive esophagitis, PPI treatment (esomeprazole 0.2–1.0 mg/kg**)** led to healing of erosions in 89% of cases after 8 weeks (Tolia et al., 2015). In the case of *Helicobacter pylori* infection, eradication therapy should consist of a PPI-based triple regimen that includes *Helicobacter pylori*-susceptible antibiotics based on antimicrobial susceptibility testing of the bacterium (Jones et al., 2017). PPIs are also recommended for the second-line eradication therapy. The dosage of PPIs used for eradication therapy in children is shown in Table 1. EoE is an immune-mediated cause of esophageal inflammation with incidence and prevalence rates in children of 5.1 cases/100,000 persons/year and 19.1 cases/100,000 persons, respectively (Arias et al., 2016). Initially, PPIs were used to differentiate EoE from PPI-responsive esophageal eosinophilia, which was thought to be related to GERD. According to the European Guidelines on EoE and the AGREE consensus statement, PPI therapy is no longer necessary for diagnosis of EoE and can be considered as part of the therapeutic management (Lucendo et al., 2017; Spergel et al., 2018). Several prospective randomized controlled trials support the efficacy of PPI therapy in inducing remission of EoE (Peterson et al., 2010; Dellon et al., 2013; Moawad et al., 2013; Vazquez-Elizondo et al., 2013), with clinic and histologic remission rates (defined as <15 eosinophils/high power field at biopsy) ranging from 33 to 57%, based on the study design and patient population. A systematic review and meta-analysis of 33 studies, including 619 EoE patients (431 adults and 188 children), reported histologic remission in about 50.5% of patients on PPI (95% CI 42.2–58.7%), with symptomatic improvement in 60.8% (95% CI 48.38–72.2%) (Lucendo et al., 2016). No significant differences were found in patients’ age, study design, and type of PPI assessed (Lucendo et al., 2016). PPIs appear to have two main favorable effects on EoE: 1) lowering acid exposure, which reduces esophageal damage; and 2) lowering eotaxin-3 levels, a Th-2 cytokine involved in eosinophil-mediated inflammation (Lucendo et al., 2017; Spergel et al., 2018). There are currently no known predictors of PPI responsiveness in EoE. Esophageal miRNAs with different expression values between PPI responsive and PPI not responsive children have been recently proposed (Cañas et al., 2020). PPI therapy is currently considered a valid first-line treatment in patients with EoE, with recommended doses of omeprazole 1–2 mg/kg twice daily or equivalent in children (Lucendo et al., 2017). Such doses should be administered for 8 weeks to assess the response. To date, few observational studies in children and adults have investigated the long-term outcomes of patients with EoE who respond to PPI therapy (Molina-Infante et al., 2015; Gómez-Torrijos et al., 2016; Gutiérrez-Junquera et al., 2018). In a recent prospective multicenter study, 78.6% of pediatric patients maintained a clinic-pathologic remission at 1-year follow-up on maintenance treatment with standard doses of esomeprazole 1 mg/kg daily (Molina-Infante et al., 2015).

**TABLE 1:** Dosage of PPIs used for *Helicobacter pylori* eradication therapy (Tolia et al., 2015).

PPI	Dosage (mg/kg/day)	Maximum daily dose (mg/day)
	Twice daily	
Lansoprazole	1.5	60
Omeprazole	1.0	40
Rabeprazole	.5	20
Esomeprazole	≥4 years old	40
	Weight <30 kg, 20 mg/day	
	Weight ≥30 kg, 40 mg/day	

PPIs, proton pump inhibitors.

### 4.2 Infants

Contrary to the indications in older children, the indications for PPI use in infants are less clear. Current consensus guidelines for treatment of GERD in children under age 1 advocate for a trial of PPI to be considered after referral to a pediatric gastroenterologist, and only after failure of 1) first-line treatments, such as thickening of feeds and avoidance of overfeeding, and 2) second-line strategies, such as a trial of cow’s milk elimination and allergy immunology consultation (due to the recognized link between cow’s milk protein allergy and GER) (Rosen et al., 2018). Esomeprazole is the only PPI approved for use in patients 1 month to younger than 12 months of age. Recommendations are consistent with available evidence that PPIs are not effective for treating symptoms usually attributed to GERD in otherwise healthy infants, such as unexplained crying, irritability, or sleep disturbance. A double-blind placebo-controlled trial of omeprazole in irritable infants with a reflux index >5% found no difference in the duration of crying between the omeprazole-treated and placebo groups, despite highly effective acid suppression in the experimental arm (Moore et al., 2003). Another large double-blind trial of 162 infants randomized to receive 4 weeks of placebo or lansoprazole, revealed the same 54% response rate in both groups, using as an endpoint >50% reduction of measures of feeding-related symptoms (crying, irritability, arching and other parameters of the Infant Gastroesophageal Reflux Questionnaire) (Orenstein et al., 2009). Similar results have also been revealed in studies of the use of pantoprazole and esomeprazole in infants (Baker et al., 2010; Winter et al., 2012). Although an improvement of symptoms during the open-label run-in period was reported, no statistically significant difference between the PPI and placebo groups was found during the stopping phase (Baker et al., 2010; Winter et al., 2012). Springer et al. (2008) analyzed the effect of lansoprazole in infants and preterm infants with GERD symptoms and reported similar profiles of changes in pHmetry parameters and gastric pH in both the treated and placebo groups. Hussain et al. (2014) studied the efficacy and safety of rabeprazole in 1- to 11-month-old infants with symptomatic GERD that was resistant to conservative therapy and/or previous exposure to anti-acid drugs. A total of 344 patients were included in an open-label phase that lasted one to 3 weeks and received rabeprazole (10 mg/day). Patients were assigned to receive placebo, rabeprazole 5 mg, or rabeprazole 10 mg in the 5-week double-blind stopping phase after caregiver-rated clinical improvement during the open-label phase, with equal improvements in symptoms and weight in all three arms (Hussain et al., 2014). Infantile reflux is frequently physiologic, and it improves on its own between the ages of 6 and 12. Caregivers reported 4159 symptoms bouts of reflux in a large study involving 186 esophageal multichannel intraluminal impedance monitoring paired with pHmetry investigations in newborns, of which only 369 could be associated with an acidic reflux event. (Garza et al., 2011). Despite the above reported evidence, there is a huge increase of prescriptions of PPIs in infants (Blank and Parkin, 2017; Levy et al., 2020; O’Reilly et al., 2020). In a recent study including over 850,000 children, 8% were prescribed a PPI before age one (Malchodi et al., 2019).

### 4.3 Other Uses

A recent 20-year observational, retrospective study analyzing PPI-related ADR reports in a national spontaneous reporting system database found more than a half (55.7%) of prescriptions being off-label. However, neither the severity nor the outcome of ADRs seemed to be linked to the label (Dipasquale et al., 2021). Chronic cough is a common off-label indication for PPI prescription in the pediatric age group, even before 1 year of age. The use of PPIs for chronic cough in children is not recommended unless there is evidence of GERD (Chang et al., 2019). In a recent retrospective study enrolling children under age 5, only 3.5% were found to have chronic cough due to GER (Chen et al., 2019). A trial evaluating 22 infants with chronic cough/wheezing found that the use of omeprazole alone did not reduce number of coughing episodes per day among those diagnosed with GER by pHmetry (Adamko et al., 2012). A larger, randomized placebo-controlled study of children with poorly controlled asthma showed no improvement in asthma control scores or pulmonary function tests when lansoprazole was added to their asthma therapy (Writing Committee for the American Lung Association Asthma Clinical Research Centers et al., 2012). Given the possibility of a link between persistent cough and GERD in children, referring them to an aerodigestive clinic might help them get better therapy.

PPIs are also widely used as stress ulcer prophylaxis, recommended in some situations to prevent upper gastrointestinal bleeding for patients admitted to the intensive care unit (Joret-Descout et al., 2017). Moreover, PPIs have showed to have benefits for reducing serum ferritin in patients with thalassemia major and intermedia (Eghbali et al., 2019).

## 5 Safety

The recent retrospective study from a national spontaneous reporting system database found 70 PPI-related adverse reaction reports in children (.01% of all database reports and 2% of all PPI adverse reaction reports, excluding literature cases), most of which were not serious or irreversible and presented with gastrointestinal (24%) and/or skin manifestations (21.3%). Notably, combination therapy (i.e., antibiotics) appeared to be positively linked with the severity of ADRs (Dipasquale et al., 2021). In terms of short-term side effects, 34% of children using PPIs experience headaches, nausea, diarrhea, or constipation (Cohen et al., 2015). In children, chronic PPI usage has been associated to an increased risk of gastrointestinal and lower respiratory tract infections, bone fractures, and allergies (De Bruyne and Ito, 2018). Although the toxicity profile of PPIs is unknown, particularly in children, pathogenetic pathways have been proposed, which are primarily connected to long-term gastric acid suppression (Table 2). Some of the most relevant literature studies on PPIs safety are summarized in Table 3.

**TABLE 2:** Main pathogenic mechanisms hypothesized for PPI-related adverse reactions in children.

System	Adverse reaction	Mechanism	
		Cause	Effect
Bone	Bone fractures	Hypochlorhydria	⁃Reduction of calcium ionization and subsequent calcium intestinal absorption
			⁃Inhibition of osteoclasts function and subsequent reduced bone resorption and remodelling
Digestive	Gastroenteritis	Hypochlorhydria	⁃Alterations in the gastrointestinal microbiome
			⁃Reduction of gastric mucus viscosity and leucocyte activity and subsequent enhanced bacterial invasion
Respiratory	Respiratory tract infections	Hypochlorhydria	⁃Invasion of microorganisms from the gastrointestinal tract into the upper and lower respiratory tract
Immune	Allergy	Hypochlorhydria	⁃Impairment of gastric and pancreatic protease activation and subsequent diminished protein processing and development of food-specific IgE
			⁃increase in mucosal permeability
			⁃Changes in the gastrointestinal microbiome

PPI, proton pump inhibitor.

**TABLE 3:** Some relevant studies on PPIs safety in children.

Concern	Details	Evidence
Infections	Increased risk of enteric infections commonly *C. difficile* driven has been observed in relation to PPI treatment in children	⁃A meta-analysis included 10,531 669 pediatric patients and found that taking PPIs raised the probability of *C. difficile* infection (Anjewierden et al., 2019)
		⁃Age over 4 years and PPI use were the independent variables for serious *C. Difficile* disease among patients with the infection in a retrospective analysis (Chang et al., 2020)
Bone fractures	Increased risk of osteoporosis and bone fractures has been observed with PPIs administration	⁃In a retrospective study of 851,631 children, of whom 97,286 (11%) were had received acid suppression <1 year of age, the use of PPI was associated with an increased risk of fracture (Malchodi et al., 2019)
		⁃Acid suppression treatment before the age of one was correlated to an earlier median age at first fracture (Wang et al., 2020)
Allergic diseases	Concerns have recently emerged regarding the use of PPIs and the development of allergy disorders	⁃A retrospective cohort study involving 792 130 children, highlighted that the risk of developing an allergic disease such as food allergies, medication allergies, anaphylaxis, allergic rhinitis, and asthma was increased in those who had received anti-acids during the first 6 months of life (Mitre et al., 2018)

PPIs, proton pump inhibitors.

### 5.1 Infections

A number of adult studies have found a possible link between PPIs and an increased risk of enteric infections, but only a few studies in children have looked into this (De Bruyne and Ito, 2018). Continuous PPI use was found to have a relative risk of 1.81 (95% CI 1.98–2.42) in a study of adults and children with acute gastroenteritis during the cold season (Vilcu et al., 2019). *Clostridium difficile* infection is the most commonly discussed infection linked to PPI use. A meta-analysis included 14 trials and 10,531,669 pediatric patients found that taking PPIs raised the probability of *C. difficile* infection by 1.33 (95% CI 1.07–1.64) (Anjewierden et al., 2019). In a more recent retrospective analysis of 124 children (1–18 years old) who had diarrhea and were positive for *C. difficile*, 49 had *C. difficile* infection and 75 had *C. difficile* colonization. Interestingly, age over 4 years (adjusted odds ratio 5.83; 95% CI 1.05–32.27) and PPI use (adjusted odds ratio 7.25; 95% CI 1.07–49.07) were the independent variables for serious illnesses among patients with the infection (Chang et al., 2020). Some studies even call for hospital regulations prohibiting the simultaneous administration of PPI and antibiotics, or for lower PPI doses, in order to reduce the risk of *C. difficile* infection (Thachil, 2008). PPIs in neonatal intensive care units have been identified as a risk factor for necrotizing enterocolitis (NEC) and sepsis, but evidence is conflicting. A prospective randomized trial showed an increased incidence of NEC in preterm infants treated with PPIs compared to the control group (Patil et al., 2017). However, a retrospective population-based study did not find any increase in NEC stage 2 and above or late onset sepsis, or mortality (Singh et al., 2016). However, most studies investigated the effect of probiotics (*Lactobacilli, Bifidobacteria* or *Saccharides*) and bovine lactoferrin to prevent NEC and sepsis. The combination of both was shown to be more effective in decreasing NEC and sepsis (Meyer and Alexander, 2017; Hagen and Skelley, 2019).

The evidence on the link between PPI usage and an increased risk of community-acquired pneumonia is mixed. In a prospective trial of PPI and ranitidine-associated infections in newborns, researchers discovered that both PPI and ranitidine usage over an 8-week period increased the risk of pneumonia (odds ratio 6.39, 95% CI 1.38–29.70) in the 4 months after enrollment (Canani et al., 2006). More recently, in New Zealand, a case-control study found no link between PPI usage and community-acquired pneumonia (*n* = 65) or lower respiratory tract infections (*n* = 566) in infants (Velasco-Benítez, 2019). The risk of respiratory tract infections in children related with PPI medications has yet to be determined.

### 5.2 Bone Fractures

Data on the risk for osteoporosis and bone fractures mainly comes from adults. In a population-based study including 124,799 cases between ages 4–29 years, a higher risk of fractures in young adults (18–29 years) on PPIs was found, but not in children (adjusted odds ratio of 1.39 vs. 1.13, respectively) (Freedberg et al., 2015). When compared to controls, children who were taken acid suppression treatment before the age of one had an earlier median age at first fracture (3.8 vs. 4.5 years) in a retrospective cohort of patients followed for 2 years. Acid suppression before the age of one and therapy for a longer period were linked to a higher risk of fracture (Wang et al., 2020). In a retrospective study of 851,631 children, 97,286 (11%) were administered acid suppression before the age of one. The use of PPI in single therapy resulted in a hazard ratio of 1.23 (95% CI 1.15–1.32) for fracture, whereas the use of a combination PPI and H2-receptor antagonist resulted in a hazard ratio of 1.32 (95% CI 1.26–1.38) for fracture (Malchodi et al., 2019).

### 5.3 Allergic Diseases

Concerns have recently been aroused about the use of PPIs and the development of allergy disorders in children, although further research is needed. Food allergies (adjusted hazard ratio 2.59, 95% CI 2.25–3.00), medication allergies (adjusted hazard ratio 1.84, 95% CI 1.56–2.17), anaphylaxis (adjusted hazard ratio 1.45, 95% CI 1.22–1.73), allergic rhinitis (adjusted hazard ratio 1.44, 95% CI 1.36–1.52), and asthma (adjusted hazard ratio 1.41, 95% CI 1.31–1.52) (Mitre et al., 2018). There appears to be a connection between prenatal PPI usage and the development of allergy (Lai et al., 2018). A meta-analysis of eight population-based studies examining anti-acids usage during pregnancy and the risk of childhood asthma symptoms indicated that mothers who used prenatal PPIs had a higher risk of childhood asthma (relative risk 1.34, 95% CI 1.18–1.52, *p* < .001) (Lai et al., 2018).

## 6 Conclusion

PPIs should only be used in children who have GERD or gastrointestinal bleeding, which should be differentiated from non-pathological GER, especially in children under the age of 1 year. In many circumstances, the risk of adverse events is minimal. However, the safety profiles of PPI usage, particularly chronic PPI use, have yet to be thoroughly defined. Current research suggests that long-term use of PPIs is linked to a variety of side effects, the most common of which are gastrointestinal events (i.e., gastrointestinal infections). Clinicians should assess if a real indication exists before prescribing PPIs, as well as the impact of PPI use on clinics and the potential harmful effects on a child’s future health.
